# Overexpression of genes involved in fatty acid biosynthesis increases lipid content in the NaHCO_3_-tolerant *Chlorella* sp. JB6

**DOI:** 10.1128/spectrum.03184-23

**Published:** 2023-12-04

**Authors:** Hongna Shang, Songsong Liu, Chenghui Xu, Shenkui Liu, Hua Liu

**Affiliations:** 1 State Key Laboratory of Subtropical Silviculture, School of Forestry and Biotechnology, Zhejiang A&F University, Lin’an, Hangzhou, China; Dominican University New York, Orangeburg, New York, USA

**Keywords:** microalgae, lipid, biosynthesis, metabolic engineering, fatty acid elongation

## Abstract

Microalgae are a potential source of biodiesel owing to their simple structure and high biomass and lipid production rates. Here, we demonstrate that fatty acid (FA) contents can be altered in *Chlorella* sp. JB6 in the presence of sodium bicarbonate (NaHCO_3_). We cloned the key genes necessary for FA *de novo* synthesis conserved in JB6, including *biotin carboxyl carrier protein* (*BCCP*), *β-carboxyltransferase* (*β-CT*), *malonyl CoA-ACP malonyl transferase* (*MCMT*), *3-ketoacyl-ACP reductase* (*KAR*), *β-ketoacyl-[acyl carrier protein] synthase I* (*KASI*), and *KASII*, and successfully transferred plasmids carrying these genes into JB6 cells. We found that overexpression of *BCCP*, *MCMT*, *KASI*, *and KASII* inhibited the growth of JB6 cells and decreased their resistance to NaHCO_3_. Notably, ectopic expression of these genes promoted very long-chain saturated FA (>20 C) accumulation. Taken together, we demonstrate that these genes are critical for JB6 growth and resistance to NaHCO_3_ and can be used for metabolic engineering.

**IMPORTANCE:**

Fatty acid (FA) contents can be altered in Chlorella JB6 in the presence of sodium bicarbonate (NaHCO_3_). Overexpression of the FA *de novo* synthesis genes inhibited the growth of JB6 cells and decreased their resistance to NaHCO_3_, but these transgenic JB6 strains could grow in a medium containing as high as 300 mM NaHCO_3_. In JB6, ectopic expression of the FA *de novo* synthesis genes increased the synthesis of very long-chain saturated FA (> 20C).

## INTRODUCTION

Fatty acids (FAs) are major components of most cellular lipids. In plants, the *de novo* biosynthesis of FAs occurs in plastids, whereas the assembly and modification of acyl lipids is accomplished in the endoplasmic reticulum (1). The FA elongation cycle includes four reactions and is governed by multiple genetic and biochemical mechanisms (2, 3). The *de novo* biosynthesis of FAs in the plastids begins with the formation of the direct substrate malonyl-coenzyme A (CoA), which is catalyzed by the acetyl-CoA carboxylase (ACCase) complex. The plant ACCase complex is composed of four catalytic subunits that assemble into the biotin carboxylase/biotin carboxyl carrier protein (BC/BCCP) and α/β-carboxyltransferase (α/β-CT) subcomplexes (4). Subsequently, malonyl-CoA and holo-acyl carrier protein (ACP) are converted to malonyl-ACP and CoA by malonyl CoA-ACP malonyltransferase (MCMT) (5). The malonyl-ACP formed is used as a two-carbon donor during the FA elongation cycle, resulting in the formation of C16 or C18 acyl-ACP (6). The enzyme β-ketoacyl-[acyl carrier protein] synthase III (KASIII) is responsible for the condensation reaction of malonyl-ACP (7, 8), and KASI and KASII are the condensing enzymes for the elongation of the carbon chain from C4 to C18 (9). In *Arabidopsis thaliana*, a reduction in the abundance of *BCCP1* transcripts affects plant growth and reduces FA accumulation (10). FA content is lower in *β-CT*-deficient mutants compared to wild-type plants, but higher in *β-CT*-overexpressing plants (11). The overexpression of *α-CT* from *Pisum sativum* in *Camelina sativa* and *A. thaliana* increases the FA content of seeds (12). Overexpression of *MCAMT* MCMT has the potential to increase the FA content and composition in dry Arabidopsis seeds (13). The *KASIII* mutation significantly reduces the total FA levels in Arabidopsis (14), and *KASI* deficiency results in a significant change in lipid composition (9).

Microalgae have received enormous attention as potential sources of biodiesel because of their high rates of biomass and lipid production (15, 16). The lipid contents in microalgae usually constitute 20%–70% of their dry weight (15). Microalgae are microscopic single cells and live in diverse ecological habitats, such as freshwater, brackish, marine, and hypersaline, with a range of temperature and pH and unique nutrient availabilities (17). The ability of microalgae to survive or proliferate under diverse environmental conditions is reflected in an exceptional variety of lipid patterns as well as in their ability to modify lipid metabolism efficiency in response to changes in environmental conditions (18, 19). Several factors currently limit the use of microalgae as a source of lipids, including the small number of species that can be grown in open systems at a low cost, the complexity of monocultures, the poor selection of new strains, inadequate extraction techniques, and low culture yields (20 – 22). The diffusion rate of CO_2_ in aqueous environments is ~10,000 times slower than in air. Most natural populations of microalgae exist in CO_2_-limited conditions (23). Exogenous CO_2_ application at an appropriate concentration promotes the growth rate of microalgae (24). Using captured CO_2_ to grow microalgae is limited by the high cost of CO_2_ capture and transportation (25). Sodium bicarbonate (NaHCO_3_) can be used as an alternative carbon source in media for microalgal cultivation to increase the lipid productivity of microalgae (26 – 28). However, the growth of most microalgae is inhibited by 15–50 mM NaHCO_3_ (29 – 32).

The *Chlorella* sp. JB6 was isolated from soda saline-alkali soil containing high concentrations of sodium carbonate (Na_2_CO_3_) and NaHCO_3_ (33). In the present study, we showed that FA content could be altered in JB6 cells in the presence of NaHCO_3_. We successfully transferred the key genes necessary for FAs *de novo* synthesis into JB6 cells and found that lipid accumulation was affected in these transgenic JB6 strains.

## RESULTS

### Lipid content was affected in JB6 cultured in medium containing glucose or NaHCO_3_

**Fig 1:**
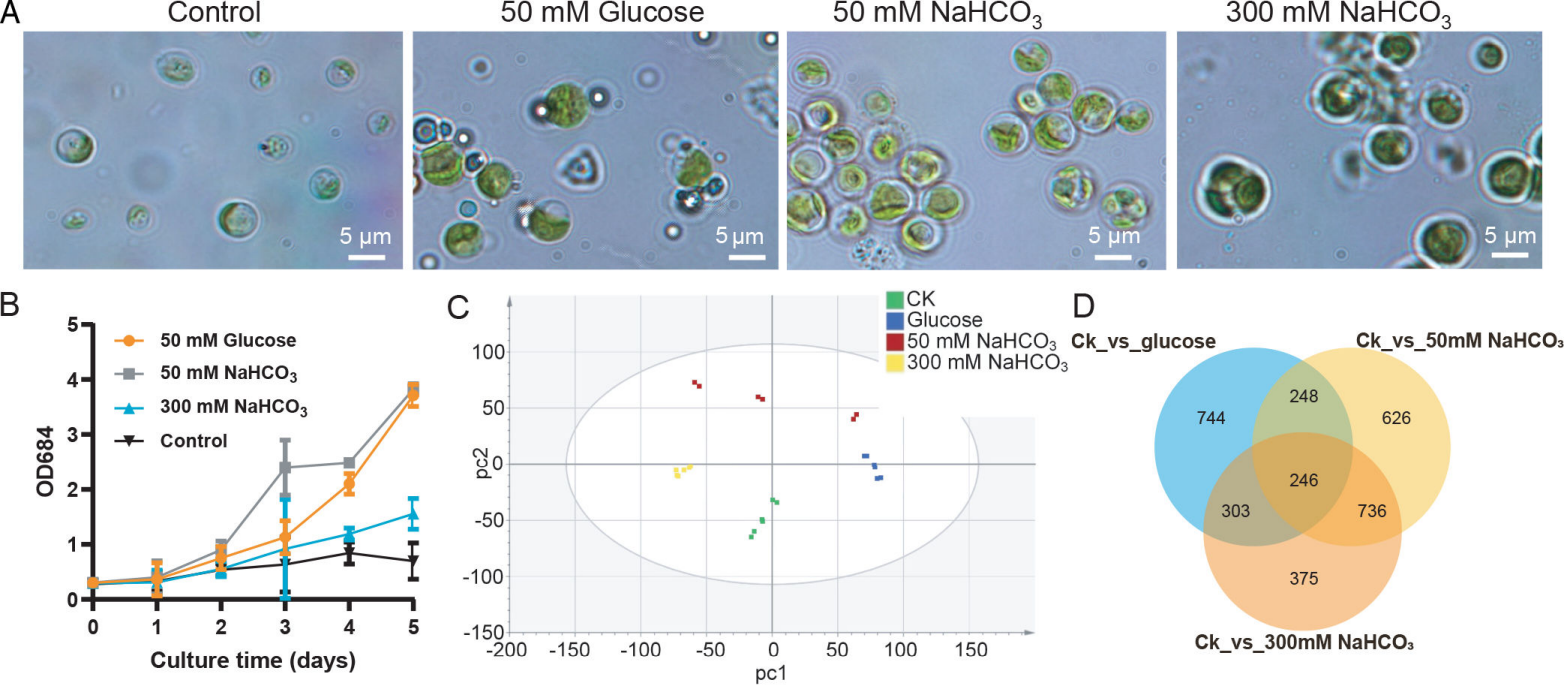
Growth and metabolite profile of JB6 cells cultured in a medium containing glucose or NaHCO_3_. (**A**) Light microscopy photographs of JB6 cells cultured in BG11 medium without carbon (control) or with 50 mM glucose, 50 mM NaHCO_3_, or 300 mM NaHCO_3_. (**B**) Growth curves of JB6 cells cultured in BG11 medium with 50 mM glucose, 50 mM NaHCO_3_, or 300 mM NaHCO_3_. (**C**) PCA of three replicates of the control and treated samples. (**D**) The Venn diagram showed the numbers of metabolites differently detected by LC-MS between treated samples and control.

Carbon sources are necessary to provide the energy and carbon skeletons for cell growth. Among various organic carbon sources such as glucose, sucrose, starch, and lactose, glucose was found to be the best in terms of biomass and lipid production in microalgae and fungi (34, 35). To evaluate the lipid production of JB6 in response to NaHCO_3_, the metabolite profile of JB6 was analyzed in the presence of glucose or NaHCO_3_. In media containing 50 mM glucose or NaHCO_3_, JB6 cells produced smaller vacuoles and bigger chloroplasts than those of JB6 cells cultured without a carbon source (control; Fig. 1A). In the absence of a carbon source, the growth of JB6 cells nearly stopped (Fig. 1B). The growth of JB6 cultured with 50 mM NaHCO_3_ was faster than that cultured with 50 mM glucose. In the presence of 300 mM NaHCO_3_, the growth of JB6 was slower than that cultured with 50 mM glucose or 50 mM NaHCO_3_ (Fig. 1B). The FA content of JB6 cells cultured with or without carbon sources was analyzed by liquid chromatography-mass spectrometry (LC-MS) in three biological triplicates. A principal component analysis (PCA) was conducted to evaluate the reliability of the LC-MS data, suggesting the variability between the control and treated samples, and in contrast to the notable differences between groups, only small differences were detected within each group (Fig. 1C). Consistently, Venn diagrams (Fig. 1D) showed that 1,541, 1,856, and 1,660 metabolites were differently detected in JB6 cells cultured in medium containing glucose, 50 mM NaHCO_3_, and 300 mM NaHCO_3_, respectively, compared to the control. In JB6 cells cultured with 50 mM glucose, saturated FAs (SFAs) and polyunsaturated FAs (PUFAs) decreased compared with the control group (Table 1). In the 50 mM and 300 mM NaHCO_3_ groups, the FAs were more abundant compared with the control and glucose groups (Table 1). For example, in the 50 mM NaHCO_3_ group, SFAs (M251T210) and PUFAs (M346T189) were elevated. In the 300 mM NaHCO_3_ group, SFA (M289T241) and PUFAs (M346T189 and M375T168) were largely increased compared with the control group (Table 1). These results suggested that the metabolite profiles were different in JB6 cells cultured in medium containing glucose or NaHCO_3_ and that NaHCO_3_ could promote FA synthesis in JB6 cells.

**TABLE 1:** Fold-changes of FA content in the treated samples compared with control^*a*^

ID	Formula	Glucose/CK	50 mM NaHCO_3_/CK	300 mM NaHCO_3_/CK
**SFA**				
M251T210	C16-0	0.231519 ± 0.039	**14.47359 ± 0.006**	–
M289T241	C16-0	–	–	**74.70378 ± 0.260**
**PUFA**				
M219T336	C16-2	–	0.041166 ± 3.83E-11	–
M217T188	C16-2	–	–	6.074456 ± 0.067
M253T188	C16-2	–	–	2.786988 ± 0.022
M265T204	C16-2	–	–	0.329375 ± 0.045
M275T301	C16-2	–	–	0.366636 ± 4.06E-10
M245T211	C18-2	–	0.213765 ± 0.041	–
M283T384	C18-2	–	4.764893 ± 0.175	4.122695 ± 0.176
M346T189	C18-2	–	**12.60419 ± 0.231**	**12.84661 ± 0.232**
M315T215	C18-2	–	3.401377 ± 0.066	3.433155 ± 0.064
M351T209	C18-2	–	2.789607 ± 0.078	–
M375T168	C18-2	–	–	**1256.176 ± 4.57E-10**
M277T194	C18-2	–	–	0.2321982.50E-07
M293T165	C18-2	–	–	0.273781 ± 0.047
M293T231	C18-2	–	–	0.282776 ± 0.060
M331T232	C18-2	–	–	0.209071 ± 0.087
M333T186	C18-2	–	–	0.296449 ± 0.058
M351T165	C18-2	–	–	0.373159 ± 0.180
M327T284	C20-2	–	–	8.980337 ± 0.077
M295T194	C16-3	0.453733 ± 0.052	–	–
M295T273	C16-3	–	0.25548 ± 0.010	0.297682 ± 0.097
M268T273	C16-3	–	0.119137 ± 1.19E-11	0.314188 ± 3.31E-11
M243T317	C18-3	–	0.363846 ± 0.052	–
M259T249	C18-3	–	2.623962 ± 1.22E-10	–
M292T198	C18-3	–	0.227346 ± 0.289	0.19733 ± 0.284
M301T316	C18-3	–	0.188766 ± 0.178	0.274139 ± 0.177
M339T223	C18-3	–	–	0.260072 ± 0.132
M259T223	C18-3	–	–	0.219619 ± 0.060
M309T215	C18-3	–	–	0.276147 ± 0.088
M317T223	C18-3	–	–	0.238898 ± 0.045

^*a*^ Boldface indicates the FAs elevated more than 10-fold in medium with 50 and 300 mM NaHCO_3_.

### Proteins encoded by FA *de novo* synthase genes were localized in the cytoplasm of JB6

**Fig 2:**
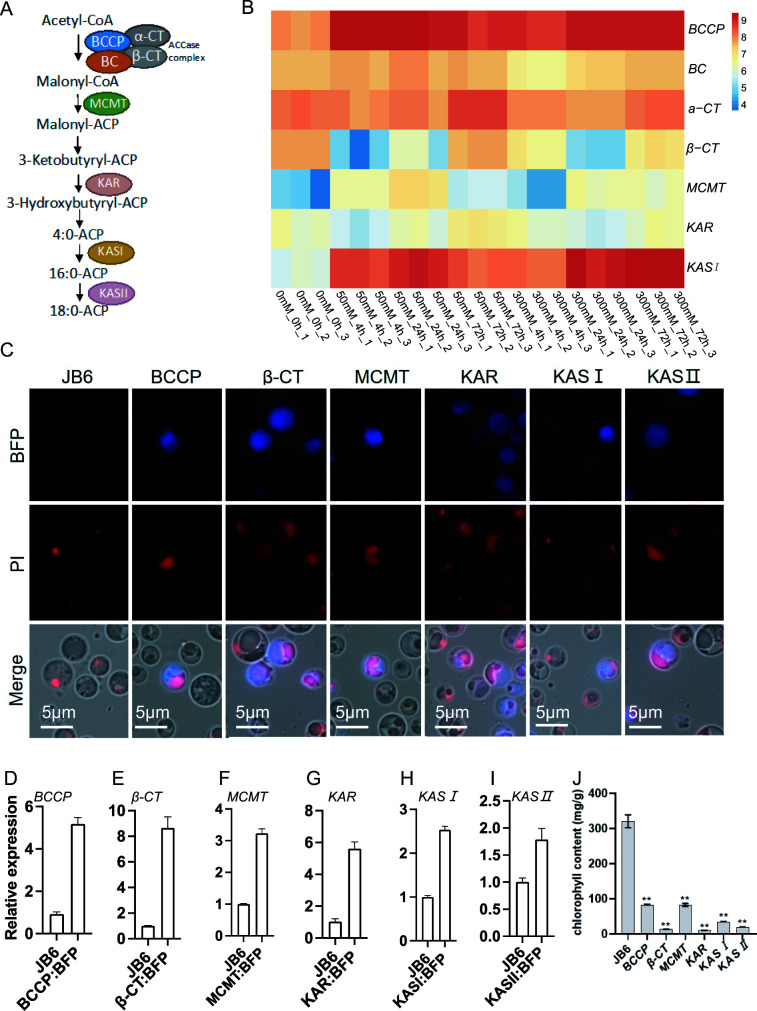
Transformation and subcellular localization of FA *de novo* synthase genes in JB6 cells. (**A**) Schematic of the biosynthetic pathway of FAs in plastid. (**B**) Different expression heatmaps of FA *de novo* synthase genes in JB6 in response to NaHCO_3_. Expression of *BCCP*, *α-CT*, *β-CT*, *MCMT*, *KAR*, and *KASI* was measured by qRT-PCR in three biological repeats. βactin was used as an internal reference gene. RNA was extracted from JB6 cells treated with 50 mM NaHCO_3_ or 300 mM NaHCO_3_ for 4, 24, and 72 h. JB6 cells treated without NaHCO_3_ (0 mM NaHCO_3_) were used as a mock. (**C**) Confocal microscopy images of wild-type JB6 and transgenic JB6 cells expressing *BFP*-tagged *BCCP*, *β-CT*, *MCMT*, *KAR*, *KASI*, and *KASII*. Nuclei were stained with propidium iodide (PI) (red). Scale bar = 5 µm. (**D–I**) Expression of *BCCP*, *β-CT*, *MCMT*, *KAR*, *KASI*, and *KASII* in the transgenic JB6 stains was measured by qRT-PCR, respectively. βactin was used as an internal reference gene. Error bars represent SD from repeats (*n* = 3). * indicates *P* ≤ 0.001; ** indicates *P* ≤ 0.0001.(**J**) Chlorophyll content in wild-type JB6 and transgenic JB6 strains. Error bars represent SD from repeats (*n* = 3). * indicates *P* ≤ 0.001; ** indicates *P* ≤ 0.0001.

To uncover the molecular mechanism of the increased FA synthesis in JB6 cells in response to NaHCO_3_ treatment, we used the sequences of *Arabidopsis* FA synthase genes as bait to screen the JB6 cDNA library and found that these genes are conserved in JB6 (Fig. 2A; Table S1). We examined the expression profile of the FA *de novo* synthase genes conserved in JB6 and found that they were differently affected in response to NaHCO_3_ treatment (Fig. 2B). The expression of *BCCP* and *KASI* was activated in response to NaHCO_3_, whereas the expression of *β-CT*, *MCMT*, and *KAR* was altered at different time points and concentrations (Fig. 2B). Additionally, the expression of KAS*II*, a critical enzyme for the elongation of the carbon chain from C16 to C18, was undetectable. Due to their different expression profiles in response to NaHCO_3_, *BCCP*, *β-CT*, *MCMT*, *KAR*, *KASI*, and *KASII* were selected for further analysis. We cloned these genes conserved in JB6, including *BCCP*, *β-CT*, *MCMT*, *KASI*, and *KASII*, and successfully transferred plasmids carrying the *blue fluorescence protein* (*BFP*)-labeled genes into JB6 cells via bombardment. Colonies, usually visible after 1–2 weeks, were retested for growth on selective media. Subsequently, the selected colonies with BFP signals were screened and used for further analysis. The synthases encoded by these genes were detected in the cytoplasm (Fig. 2C), which is consistent with the finding that the *de novo* synthesis of plant FAs occurs in the cytoplasm. The expression levels of these genes were increased in the transgenic JB6 stains (Fig. 2D through I). Additionally, compared with wild-type JB6, the transgenic JB6 strains exhibited decreased chlorophyll fluorescence (Fig. 2J), which may have resulted from the disturbances in FA content (9, 11).

### Growth of JB6 cells was affected by overexpressing FA *de novo* synthase genes

**Fig 3:**
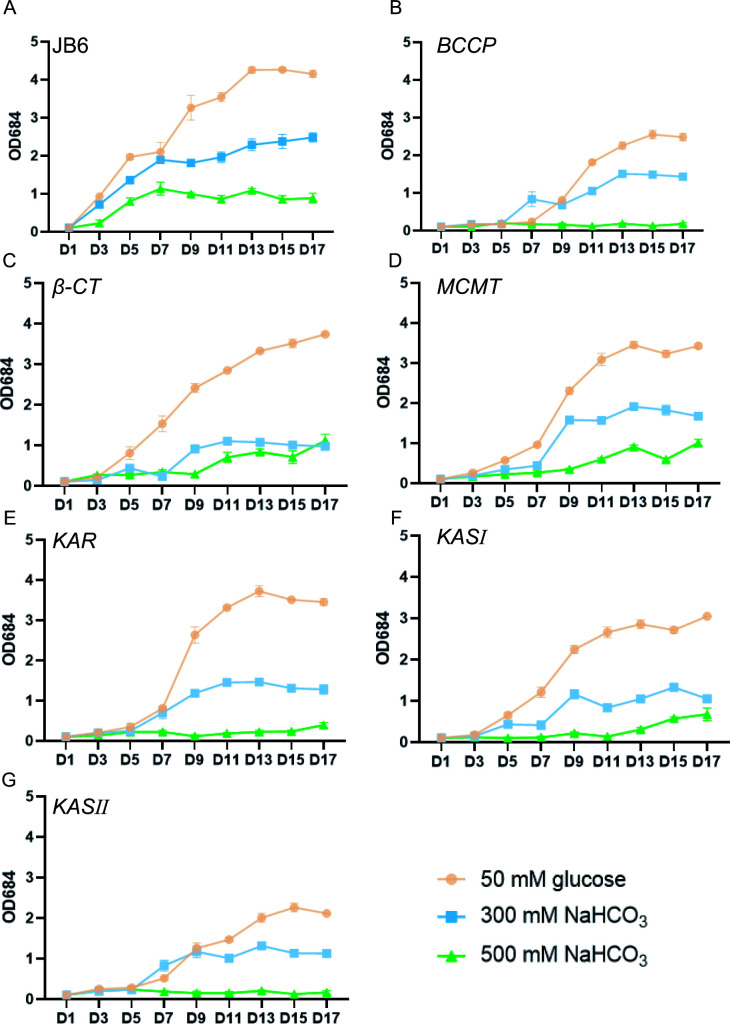
Growth of JB6 cells overexpressing FA *de novo* synthase genes. Growth curves of wild-type and transgenic JB6 strains cultured in BG11 with 50 mM glucose, 50 mM or 300 mM NaHCO_3_. Error bars represent SD from repeats (*n* = 3).

A high rate of biomass was a critical factor in evaluating the microalgae used for biodiesel production. To evaluate the growth of the transgenic JB6 strains in response to NaHCO_3_ treatment, we measured the cell density of the transgenic strains cultured in medium containing either 50 mM glucose, 300 mM NaHCO_3_, or 500 mM NaHCO_3_ (Fig. 3). The cell density of JB6 overexpressing *β-CT* and *KAR* cultured with 50 mM glucose resembled that of wild-type JB6 (Fig. 3C and E). The cell density of JB6 overexpressing *BCCP*, *MCMT*, *KASI*, and *KASII* cultured with 50 mM glucose was lower than that of wild-type JB6 (Fig. 3B, D, F, and G), suggesting that overexpression of these genes affected the growth of JB6 cells. All the cell densities of the transgenic JB6 strains cultured with 300 mM NaHCO_3_ or 500 mM NaHCO_3_ were lower than those of wild-type JB6, indicating that the ectopic expression of these genes decreased the resistance of JB6 cells to NaHCO_3_ treatment. In contrast to the growth of other microalgae, which is normally inhibited by 15–50 mM NaHCO_3_ (29 – 32), these transgenic JB6 strains could grow in a medium containing as high as 300 mM NaHCO_3_.

### Lipid content was affected by overexpressing FA *de novo* synthase genes

**Fig 4:**
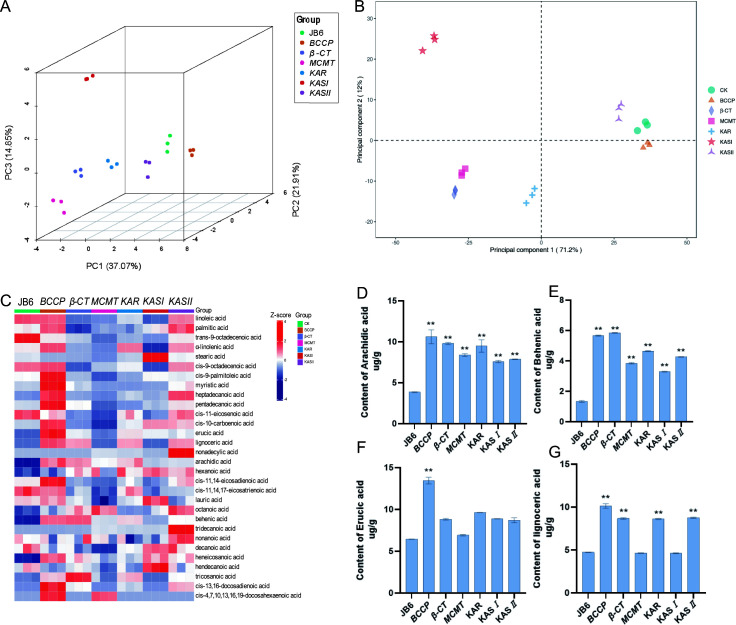
FA content was detected by GC-MS in wild-type and transgenic JB6 strains. (**A**) PCA of three replicates of wild-type and transgenic JB6 strains. (**B**) PLS-DA of FAs in transgenic JB6 strains in positive ion mode.(**C**) Clustering heat map of FAs between wild-type and transgenic JB6 strains. (**D**) Content of arachidic acid, behenic acid, erucic acid, and lignoceric acid in wild-type and transgenic JB6 strains. Error bars represent SD from repeats (*n* = 3). **indicates *P* ≤ 0.0001.

To explore the FA contents of transgenic JB6 cells in response to NaHCO_3_ treatment, gas chromatography-mass spectrometry (GC-MS) was conducted by using JB6 cells cultured with 100 mM NaHCO_3_ in three biological triplicates. PCA and partial least squares-discriminate analysis (PLS-DA) assays showed a clear separation between wild-type JB6 and the transgenic JB6 strains (Fig. 4A and B). Consistently, a dramatic alteration in FA content was detected in the transgenic JB6 strains compared to the wild-type JB6 (Fig. 4C; Table 2). Monounsaturated FAs (MUFA) and PUFA content were unchanged or decreased in most transgenic JB6 strains, whereas very long-chain SFA (>20 C) content was increased in the transgenic JB6 strains (Table 2). FA content was increased in JB6 overexpressing *BCCP* and *KASII* (Fig. 4C; Table 2). Additionally, overexpression of *BCCP* enhanced arachidic acid, behenic acid, erucic acid, and lignoceric acid production by 2.63-, 4.31-, 2.09- and 2.14-fold, respectively (Fig. 4D though G). Overexpression of *β-CT, KAR* and *KASII* did not significantly affect erucic acid production, but enhanced arachidic acid, behenic acid, and lignoceric acid production (Fig. 4D, E, and G). Overexpression of *MCMT* and *KASI* enhanced the production of arachidic acid and behenic acid (Fig. 4D and E). Taken together, these genes have the potential to be used for metabolic engineering to increase lipid production in microalgae. However, the production of MUFA and PUFA was not improved in most transgenic JB6 strains. Hence, the genes involved in the assembly and modification of acyl lipids should be necessary to be analyzed to improve FA quality.

**TABLE 2:** FA content in wild-type and transgenic JB6^*a*^

	JB6	BCCP	Β-CT	MCMT	KAR	KASI	KASII
**SFA**							
C13-0	1.334 ± 0.044	1.302 ± 0.033	1.474 ± 0.097	**3.491 ± 0.122**	**2.846 ± 0.188**	**2.295 ± 0.133**	**17.605 ± 0.547**
C14-0	22.298 ± 0.177	**42.015 ± 1.099**	23.373 ± 1.465	19.190 ± 0.163	19.108 ± 0.032	19.750 ± 0.082	26.216 ± 1.336
C15-0	14.494 ± 0.218	**36.418 ± 0.753**	17.565 ± 0.275	5.587 ± 0.057	9.549 ± 0.044	13.111 ± 0.734	19.268 ± 0.153
C16-0	1,373.659 ± 25.727	1,535.073 ± 12.448	1,121.278 ± 16.531	1,254.170 ± 15.616	1,250.438 ± 40.704	1,374.645 ± 40.752	1,551.272 ± 39.184
C17-0	16.606 ± 0.520	**40.716 ± 1.531**	18.377 ± 0.349	15.567 ± 0.283	21.138 ± 1.069	18.916 ± 0.838	**37.545 ± 0.234**
C18-0	462.923 ± 2.885	429.019 ± 0.803	423.656 ± 1.468	398.003 ± 0.387	448.943 ± 2.946	**698.943 ± 0.619**	474.912 ± 3.514
C19-0	4.588 ± 0.296	2.772 ± 0.061	2.902 ± 0.062	**16.271 ± 0.271**	**12.933 ± 0.593**	4.391 ± 0.253	**120.718 ± 0.332**
C20-0	3.852 ± 0.046	**10.603 ± 0.850**	**9.789 ± 0.136**	**8.374 ± 0.166**	**9.484 ± 0.760**	**7.588 ± 0.145**	**7.858 ± 0.031**
C21-0	0.365 ± 0.042	**1.055 ± 0.035**	**0.874 ± 0.073**	**0.703 ± 0.078**	**0.752 ± 0.047**	**0.993 ± 0.075**	**0.878 ± 0.069**
C22-0	1.328 ± 0.060	**5.665 ± 0.041**	**5.847 ± 0.024**	**3.851 ± 0.046**	**4.645 ± 0.029**	**3.289 ± 0.024**	**4.262± 0.017**
C23-0	0.132 ± 0.015	**1.267 ± 0.040**	**2.427 ± 0.113**	**0.699**	**1.320 ± 0.085**	**0**	**0.964 ± 0.036**
C24-0	4.732 ± 0.026	**10.125 ± 0.271**	**8.680 ± 0.095**	4.647 ± 0.026	**8.609 ± 0.068**	4.629 ± 0.056	**8.761 ± 0.068**
**MUFA**							
C16-1	25.276 ± 0.318	**55.646 ± 0.172**	18.600 ± 0.170	6.362 ± 0.033	16.233 ± 0.240	26.471 ± 0.319	23.678 ± 0.389
C18-1	314.309 ± 0.776	331.848 ± 0.169	171.058 ± 2.244	226.522 ± 0.364	248.917 ± 0.024	162.913 ± 0.067	299.913 ± 0.497
C19-1	7.270 ± 0.028	10.331 ± 0.120	6.461 ± 0.054	5.034 ± 0.039	7.616 ± 0.095	8.941 ± 0.018	8.587 ± 0.199
C22-1	6.441 ± 0.010	**13.457 ± 0.397**	8.825 ± 0.103	6.917 ± 0.088	9.630 ± 0.016	8.884 ± 0.009	8.714 ± 0.288
**PUFA**							
C18-2	2,446.604 ± 38.600	2,374.095 ± 24.803	1,242.657 ± 11.451	1,345.762 ± 11.878	1,673.071 ± 13.327	1,160.449 ± 45.000	2,216.836 ± 15.467
C18-3	747.963 ± 2.201	953.705 ± 4.295	586.941 ± 2.428	526.438 ± 1.528	877.315 ± 2.195	366.090 ± 0.721	844.630 ± 3.017
C22-6	2.201	2.364 ± 0.115	0	1.795 ± 0.114	0	0	0

^*a*^ Boldface indicates the FAs are significantly elevated in the transgenic JB6. Each value represents the mean ± SD (n = 3).

### Expression of FA *de novo* synthase genes was affected in the transgenic JB6 strains

**Fig 5:**
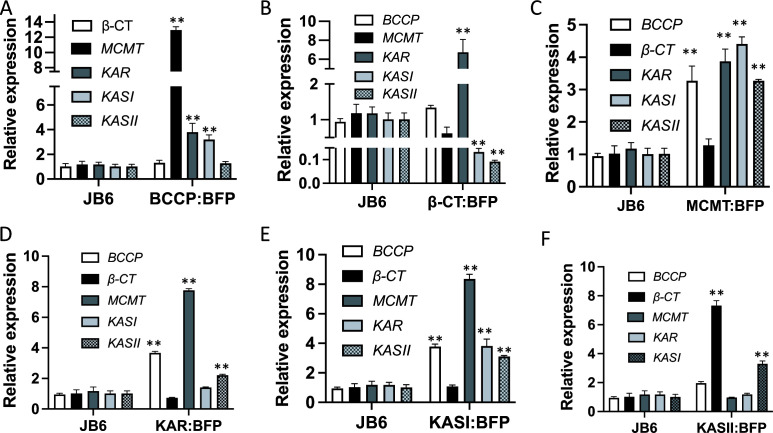
Expression of *BCCP*, *β-CT*, *MCMT*, *KAR*, *KASI*, and *KASII* in the transgenic JB6 strains. Expression of FA *de novo* synthase genes was measured by qRT-PCR, respectively. βactin was used as an internal reference gene. Error bars represent SD from repeats (*n* = 3). * indicates *P* ≤ 0.001; ** indicates *P* ≤ 0.0001.

To evaluate the expression of the genes involved in FA biosynthesis in the transgenic JB6 strains, qRT-PCR assay was performed in the wild-type and transgenic JB6 stains. Generally, expression of most genes involved in FA biosynthesis was enhanced in the transgenic JB6 strains (Fig. 5). Compared with the wild-type JB6, the transcript levels of *MCMT, KAR,* and *KASI* were significantly increased in JB6 overexpressing *BCCP* (Fig. 5A). In JB6 overexpressing *β-CT*, the transcript level of *KAR* was increased*,* while *KASI* and *KASII* were decreased (Fig. 5B). In JB6 overexpressing *MCMT*, the transcript levels of *BCCP*, *KAR*, *KASI*, and *KASII* were increased (Fig. 5C). In JB6 overexpressing *KAR,* the transcript levels of BCCP, *MCMT,* and *KASII* were increased, with a subtle increase in the expression of *KASI* (Fig. 5D). In JB6 overexpressing *KASI,* the transcript levels of BCCP, *MCAT, KAR,* and *KASII* were increased (Fig. 5E). In JB6 overexpressing *KASII,* the transcript levels of *β-CT* and *KASI* were increased (Fig. 5F). These data suggested that the alteration in FA contents in the transgenic JB6 stains resulted from the altered expression of genes involved in FA biosynthesis.

## DISCUSSION

Microalgae have attracted considerable interest worldwide because of their potential for a wide range of applications in the renewable energy, biopharmaceutical, and nutritional industries (36, 37). The selection of new strains that can be cultivated to yield high biomass, high lipid content, and good biodiesel qualities at a low cost will promote the use of microalgae as a source of lipids. In this study, we demonstrated that *Chlorella* sp. JB6 can be cultured in a medium containing NaHCO_3_, an economical carbon source, to increase its FA content. We successfully transferred the genes involved in the FAs *de novo* synthesis pathway into JB6 cells and found that lipid accumulation and quality were improved in the transgenic JB6 strains overexpressing *BCCP* and *KASII*.

Carbon sources are necessary to provide the energy and carbon skeletons for cell growth. Exogenous CO_2_ application at an appropriate concentration promotes the growth rate of microalgae (24). Using captured CO_2_ to grow microalgae is limited by the high cost of CO_2_ capture and transportation (25). NaHCO_3_ can be used as an economical carbon source to improve microalgal biomass and lipid content (26 – 29, 31). The growth of most microalgae is inhibited by 15–50 mM NaHCO_3_ (29 – 32). In contrast, JB6 cells had vigorous growth at 50–400 mM NaHCO_3_ (33). Among various organic carbon sources such as glucose, sucrose, starch, and lactose, glucose was found to be the best in terms of biomass and lipid production in microalgae and fungi (34, 35). In the medium containing NaHCO_3_, JB6 cells produced more FAs than those cultured with glucose, suggesting that NaHCO_3_ could promote FA synthesis in JB6 cells. We had transferred the genes involved in the FAs *de novo* synthesis pathway into JB6 cells. The transgenic JB6 stains exhibited reduced biomass compared to wild-type JB6 when they were cultured in a medium with a high concentration of NaHCO_3_. In contrast to the growth of other microalgae, which is normally inhibited by 15–50 mM NaHCO_3_ (29–32), these transgenic JB6 strains could grow in a medium containing as high as 300 mM NaHCO_3_. By measuring the FA content of transgenic JB6 stains, we found that very long-chain SFA (>20C) content was increased in the transgenic JB6 strains, which could result from the fact that these genes are responsible for FA elongation (1, 4, 6, 9). However, the production of MUFA and PUFA was unchanged or decreased in most transgenic JB6 strains. Hence, the genes involved in the assembly and modification of acyl lipids should be necessary to be analyzed to improve FA quality. Taken together, these genes identified in this study are critical resources for JB6 growth and resistance to NaHCO_3_ and have the potential to be used for metabolic engineering to increase lipid production in microalgae.

## MATERIALS AND METHODS

### Microalgae training and culture conditions

*Chlorella sp.* JB6 was isolated from the extreme saline soil of the Songnen Plain in China (33). JB6 cells were cultured in BG-11 medium supplemented with glucose or NaHCO_3_. JB6 cells were cultured at a temperature of 24°C under a 16 h/8 h (light/dark) photoperiod with 80 µmol photons m^−2^ s^−1^ fluorescent white light.

### Growth evaluation

To monitor JB6 cell growth, 200 µL of JB6 cells (initial cell concentration, 10^6^ cells mL^−1^) was cultured in 5 mL of fresh BG11 with different concentrations of NaHCO_3_ or 50 mM glucose for 15 d. JB6 cells cultured without carbon were used as a control. The OD680 of the algal solution was measured by NanoDrop One (Thermo Fisher Scientific).

### Gene cloning and plasmid construction

Six FA *de novo* synthase genes, including *BCCP* (AT5G15530.1), *β-CT* (ATCG00500.1), *MCMT* (AT2G30200.1), *KAR* (AT1G63380.1), *KASI* (AT5G46290.3), and *KASII* (AT1G74960.2), were identified from Arabidopsis and used as query sequences to perform BLAST in the JB6 cDNA library. BFP gene was inserted into the *BamH*I site of pLM006 (Biovector) by using the In-Fusion Cloning Snap Assembly Master Mix (Clontech), generating pLM006:*BFP*. The full-length cDNA of FA *de novo* synthase genes identified from the JB6 cDNA library without stop codon was cloned into the *BamH*I site of the pLM006:*BFP* by using the In-Fusion Cloning Snap Assembly Master Mix. Information about the candidates identified in JB6 are listed in Supplementary File 1 and Table S1. Primers used for cloning are listed in Table S2.

### Transformation and subcellular localization

JB6 were cultured in BG11 liquid medium for 3–4 d at 23 ± 1°C under a 16:8 h light/dark cycle at 80 µmol photons m^−2^ s^−1^ fluorescent white light. The cultured JB6 was isolated from liquid medium and then coated on the surface of BG11 solid medium and incubated at room temperature for another 3–4 d until the algae were used for bombardment (Bio-Red, PDS-100). The transformed JB6 cells were cultured in BG11 solid medium in darkness for 24 h, and thereafter, the cells were harvested and cultured in the solid selection medium supplemented with hygromycin (20 mg/mL). Colonies were usually visible after 1 to 2 weeks, and then the transformants with BFP were screened by a confocal fluorescence microscope (Carl Zeiss, LSM 880) and used for the next screen.

### Expression analysis

Total RNA was isolated from JB6 cells with TRIzol (Invitrogen). A cDNA library was prepared using PrimeScript Reverse Transcriptase (TaKaRa). Quantitative reverse transcription PCR (qRT-PCR) was carried out using a StepOne Real-Time PCR System (Applied Biosystems) and the SYBR Premix Ex Taq (TaKaRa). The primers used for qRT-PCR are listed in Table S3.

### FA composition analysis

Lipids were extracted from the stationary-phase cultures (250 mL each) of wild-type and transgenic JB6 strains. Quantification of FAs was carried out by GC-MS. The lyophilized sample was weighed at 20 mg, and 150 µL of methanol solution, 200 µL of methyl tert-butyl ether solution, and 50 µL of 36% phosphoric acid solution were added. The sample was shaken at room temperature for 3 min, sonicated in ice water for 10 min, and centrifuged at 4°C and 12,000 r/min for 5 min. Two hundred microliter of supernatant was pipetted into a 2-mL centrifuge tube, 20 µL of internal standard working solution was added, transferred to the concentrator, and evaporated. Add 200 µL of boron trifluoride methanol solution, vortex for 3 min, and keep in an oven at 60°C for 30 min; cool to room temperature. Add 500 µL of hexane solution and 200 µL of saturated sodium chloride solution accurately; shake for 3 min, and centrifuge at 4°C and 12,000 r/min for 5 min. Pipette 100 µL of hexane layer solution for onboard analysis and store in the refrigerator at −20°C.

### LC-MS analysis

Chromatographic separations were performed using an ultra-performance liquid chromatography system (SCIEX). An XBridge BEH C18 column (3.5 µm, 2.1 mm × 100 mm; Waters, UK) was used for the reversed phase separation. The column oven was maintained at 50°C. The flow rate was 0.3 mL/min, and the mobile phase consisted of solvent A (water + 0.1% formic acid) and solvent B (acetonitrile + 0.1% formic acid). Gradient elution conditions were set as follows: 0–0.5 min, 5% phase B; 0.5–5 min, 5% to 80% phase B; 5–7 min, 80%–100% phase B; 7–8 min, 100% phase B; 8–8.1 min, 100%–5% phase B; 8.1–10 min, 5% phase B. The injection volume for each sample was 10 µL.

## SUPPLEMENTAL MATERIAL

Supplemental material spectrum.03184-23-s0001.pdf Tables S1 to S3.

ASM does not own the copyrights to Supplemental Material that may be linked to, or accessed through, an article. The authors have granted ASM a non-exclusive, world-wide license to publish the Supplemental Material files. Please contact the corresponding author directly for reuse.
