# Supplementary material for: Overexpression of genes involved in fatty acid biosynthesis increases lipid content in the NaHCO3-tolerant Chlorella sp. JB6
Source: Microbiol Spectr. 2023 Dec 4;12(1):e03184-23. doi: 10.1128/spectrum.03184-23 (PMC10783073; doi:10.1128/spectrum.03184-23)
Supplement: Supplemental material — Tables S1 to S3. [file spectrum.03184-23-s0001.pdf]

Supplementary File 1

jb602c0162900.1
ATGCAGGCAGCCATCACGGAGCGTGCGTTTGCGGTCAGGCCAGCCGGCCGCGCTGCTGTGCAGCCCCGGCCGCTCCCCTCGGCATCGACGTG
CCCCAAGGCTCGCTCGGTGCAAGCACGGGCTGCAGCTGAGGAGGTCAAGGCAGCCAGCACCAATGGGGCGCCCCTCAAGCCAGGCACGGCAG
CCCTGGACTTCGACGAGTTGACGGAGCTGATTAAGATGGTGCACTCCACCGACATTGTGGAGCTGGAGCTGAACAGCAAGAAGTTCAAGTTGTCT
GTGAAGAAGAAGGAGGCGCTGGAAGCGTCAGAGCCGCAAATCATTCATATGAGCGCCCCCGCAGGCTACGCAGCGCAGGCGCCAGCGCCCGCG
CCAGCTGCCCCCGTTGCTGCTGCTACCACCGCTGCGCCCGCCGCACCCGCTGCGCCTGCACCTGCCGCAGCAGCACCGGCTGCGGTGGATGG
CCTGGAGGTGGTGTCGCCTATGGCAGGCACCATGTACCGTTCCCCAGCTCCGGGGGAGCCCGTGTTTTGCAAGGAGGGTGACCACGTCAACAAG
GGCGACGTCATTTGCATCATTGAGGCCATGAAGCTGATGAACACCCTGGAAGCGGAGGTTTCGGGAACCGTGGTCAAGGTCATGGCAGAGAATGG
CGACTCCGTGCTGCCGGGCCAGCCCCTGATGATCATCCGCCCTTGA
jb604c0327600.1
ATGAGCAGCAACGCAGGAGAGCTGGCGCACGCACTGGACGATCTGCCTCCGTATCGACCAGGCAGCAATCCCAAGTGGGAGCGAGTTGAGGCG
GCCAAACGGGAGGCAGCCCTGGGCGGCGGAGAGGCTCGTATTGCCAAGCAGCACAAGCAGGGCAAGCTGACAGCACGCGAGCGGCTCCAGCT
GCTGTTTGACCCAGGCAGCTTCAAGGAGGCTGGCGCCCTGGTGCAGCACCGTCGCAACGACTTTGACATGGACCGCCAGCAGCCCTTTGGAGAC
GGCGTGGTGACCGGCAGCGGCAAGGTGTTTGGCCGCCCGGTGTTTGCCTTCAGCCAGGACTTTACAGTGTTTGGTGGCTCGCTGTCGGAGTCC
CACGCACTGAAGATATGCCGGCTTATGGACCGCGCTGTGGCGGCAGGGGCTCCAGTCGTGGGGCTGAACGACAGTGGCGGCGCCCGCATTCAG
GAGGGTGTGATGTCACTGGCTGGGTATGCTGAGGTGTTCCAGCGAAACGTGGACGCTTCAGGGGTTGTGCCGCAGCTGTCGCTGGTGATGGGG
CCGTGTGCGGGTGGTGCGGTTTACTCACCGGCCCTCACAGACTTCACCTTCATGGTACGTCACTCCAGCTACATGTTTCTGACTGGTCCCGAGGT
GGTGAGGAGCGTCACCCGCGAGGAGGTGACGCAGGAGCAGCTGGGGGGCGCTGGAACGCACACCACCAAGTCAGGTGTGGCGCATGGTGCCT
TCGACAATGAGTTAGATGCTCTGGCTGGTGTGCGGGAGCTGCTCTCCTTCCTCCCTCTTTCCAACAGGGACAAGCTGCTGCAGGCAAGTCGACCA
GGCGCTCTGCTAGTTACCCCTTGCACCGACCCAACTGACAGGCAGTGCCCGTACCTGGATTACGTTGTGCCTGGCAGCGAGCTGGAGGCCTACG
ACATGCTTGGAGTGGTCCAGCAGGTGGTGGACGACGGCCAGGTGCTGGAGATTATGCGGGAGTATGCTCGCAACATGATAGTAGGCTTCGCACG
CCTTGAGGGGCGCACCGTGGGCATTGTGGCCAACCAACCGGCTGTGCTGGCAGGCTGCCTGGACATCGATGCCTCAGTCAAGGCTGCTCGCTTT
GTGCGCTTCTGCGATGCGTTCAACATCCCCCTGCTCACATTTGTCGACGTGCCCGGCTTCTTGCCTGGCACTGCCCAGGAGTATGGCGGCATCAT
CCGGCACGGCGCCAAGCTGCTGTATGCTTATGCCGAGGCCACAGTGCCCAAGCTGACTGTGATCACGAGGAAGGCCTTTGGTGGCGCCTACGAC
GTGATGTCCAGCAAGCACTTGCGGGGTGACGTCAACCTGTCGTGGCCCACCGGCCAGATTGCTGTGATGGGCGCCAAGGGTGCCGTGGAGATC
CTGTTCAGGGGCAAGGACACCGACATGAAGCAGCAGGAGCAGGAGTATGAGCTCAAGTTCAGCAACCCGTTCCAGGCGGCCAAGGTCGGGTTCA
TTGATGATGTGATCCTGCCCCGCACGACGCGCCAGCGGCTGTGCCAAGAGCTGGAGGTGTTGGCAGACAAGAAGGTGTGGCGCCCGCAACGCA
AGCATGGCAATATTCCACTGTAA
jb613c0887700.1
ATGCAGGCCGCCGCGATTCAGAGTGTAGTGCGAGGGTTCGCGGTCCGCCAGGGCGCCGGCAGGCACGGCCGCAGGCAGGTGGCGGTCAAGG
CCAGCTCTGACGCCAAGTTTGCGGACTACAAGCCCAAGGTGGCATTCTTCTTCCCCGGCCAGGGTGCTCAGACGGTGGGCATGGGCAAGGAGGT
GTCTGAGTCTGTGCCTGCCGCCAAGCAGCTCTTTGAGCGCGCCTCTGACATCCTGGGCTACGACCTGCTGGCCGTGTGCGGTGAAGGCCCCAAG
GAGCGGCTCGACTCCACCGCTGTCAGCCAGCCCGCCATCTACGTCGCCTCGCTGGCAGCCCTGGAGAAGCTCAAGGCTGAGCAGGGCCCCGAG
GCTGCCGACGCTGCAGATGTGTGCTGCGGGCTGTCACTGGGGGAATACACGGCGCTCACCCATGCGGGTGCCTTGTCATTTGAAGACGGGGTGA
GGCTGGTGAAGATCCGGGGCGAGAGCATGCAGGCAGCGGCGGACGCACAGCCCTCGGGCATGGTCAGCGTGATTGGTCTGTCAGCCGACAAG
GTGGCCGAGCTGTGTGAGGCTGCCAGCAAGGAGGTGCTGGAGGGGCAGGGTGTGCGTATAGCCAACTACCTCTGCAACGGCAACTACGCGGTC
AGCGGCGGCATCGCGGGTTGTGAGGCGCTGGAGGGCATGGCCAAGAGCTTCAAGGCCAGGATGACTGTGCGGCTGGCGGTGGCGGGTGCCTT
CCACACCGACTTCATGGCGCCAGCCAGGGAGAAGCTGCAGGAGGCGCTGGCGCGCACTACCATCCGGGAGCCCCGCATCCCGGTGGTGTCCAA
CGTGGATGCCGCCCCCCACAGCGACCCAGATGTGATCAAGTCCATCCTGGCGCAGCAGCTGACGGCACCTGTGCAGTGGGAGACCAGCATCAGG
ACGCTGCTGGACAGGGGCTTGGAGCGCAGCTACGAGATCGGGCCAAACAAGGTCATTGCCGGCATCATGAAGCGCATTGACAAGACCCACCCCC
TGGAGAACATCACTGTGTGA
jb601c0096200.1
ATGTCGAAGCTTGTCGGGAAGGTCGCCCTGATAACGGGAGGGGCACGAGGCATTGGGCTGGCCACCGCGCAAGCGCTTGGCAAGGAGGGGGC
AAAGGTGGTGGTGGCAGACATTGATGCAGATGCTGTGCGACAGGCGGCAACGCAGCTGCAAGCAGAGGGCGTGGAAGCATGCAGCATTGCGTG
CGATGTTGGGGACAAGGCCCAAGTGCATGCTGCGGTGGCCGAGGCAGTGGGCCGCTACGGCGGTCTTGACATCGCTGTTGCCAATGCGGGCAT
AGTGCGCAGCGCAGACTTTCTCGACATGAGCGAGGCAGACTTCGACGCCGTGCTGCGAGTCAACCTCAAAGGCACCTTCTTGACGGGGCAAGCG
GCAGCAAAGCAGATGGTTGCGCAGGGGCGTGGCGGCGTGATTGTCAACATGAGCTCAGTCAACGGCATCACAGCTATACCGACGATTGCTGCGTA
CAATGCGAGCAAGGGTGGCATTGACAACCTCACTCGCTGCATGGCTCTGGCGCTGGCGCCCCACAAGATACGAGTCAACGCTGTGGGCCCGGGT
TCAATCATGACAGATGTGCTGCAGTCGGTGGTGACAGACAAGGAGGCCATGGGCAGGGTGCTGTCCAGGACCCCTATGCTGCGTGTGGGGCAGC
CCAGCGAGGTTGCTTCCGTGGTGTGCTTCCTGTCGTCCAATGACTCCAGCTACATGACCGGCCAGACGCTGTACGTGGATGGCGGCCGCCTGGC
CCTCAACTACACCGTCAAAGTGCCGGAGGAAGCCATGTAA
jb607c0563500.1
ATGTCACCAGCGCGTGTGCCGGAGGCTTTGAAGGCCGGGCTGTCGCTCAGCGCGACAAGGGCTGCCCGCCCAAGATCGGTGCTGGTGCAAGCC
GCCGCCGCGCAGCGCCGCACCGACCCCAAGAAGCGGGTCGTCATTACTGGCATGGGCCTCGCATCCTGCTTTGGAAACGACGTTGATCACTTTTA
CAACCAGCTGCTGGAGGGAGTGAGCGGTGTGGACTACATTTCCCGGTTTGATGCCAGCAACTTCCCCACCAAGTTTGCAGCACAGATCAAAGACT
TTGATGTTGGTGACCTGGTTGACAAGAAGAACGCACGTCGCTATGACGACTGCCTCTCTTACACCATGGTCGCCTCGAAAAAGGCCCTCCAAGCTG
CTGGCTTGGAGAAGCATGCCGACCCGGAAGGCCACGGGGCGCTGGACAAGACCCGTGTTGGTGTGCTGGTGGGCAGCGGCATGGGCGGTCTG
TCTGTGTTCCAGGATGGTGTGAAGGCGCTCGTGGAGAAGGGCCACAAGAAGATCACGCCCTTCTTCATCCCATACGCAATCACCAACATGGGTGG
CGCCCTGTTGGCTATCGACCAGGGGTTCATGGGGCCGAACTACTCCATTTCGACCGCATGCGCCACCGCAAACTACGCATTTGTGTCAGCTGCCA
ACCACATCCGTAATGGCGATGCTGATGTGATGGTCGCTGGCGGCAGTGAGGCACCCATCATCCCTGTAGGCCTCGGCGGTTTTGTGGCATGCAGA
GCTCTTAGTCAGCGCAACGATGAGCCGCAGCGGGCGTCGCGCCCTTGGGACGATGGGCGGGATGGGTTTGTGATGGGCGAGGGCGCGGGCGT
CCTGGTCATGGAGAGCCTGGAGCATGCACAGAAGCGGGGTGCCACCATCATTGCGGAATACTTGGGCGGCGCTGTCACCTGCGACGCGCACCAC
ATGACGGATCCCCACCCCGATGGCCTGGGCGTGTCCACATGCATCGAGCTTGCGCTCAAAGACTCTGGCATTGAGCGGGACGAGGTGAACTACAT
CAATGCTCACGCGACCAGCACCCTGGTGGGGGACAAGGCAGAGGTGAAGGCGATCAAGAAGGTGTTCACTGATCTGTCGCACATCAAGATGAAC
GCCACCAAGAGCATGATCGGGCATTGCCTGGGAGCAGCCGGCGGCATGGAGGCCATTGCCACCATTCAGGCCATGCGAACGGGCTGGGTCCAC
CCCACCATCAATCACGAGTCACCAATCGAGGAGGTGGACGGCATTGATGTTGTTGCCAACACGAAGAAGCAGCATGATGTGAATGTAGGCATCAGC
AACAGCTTCGGTTTTGGTGGCCACAACAGTGTGTGCGCGTTTGGAAAGTACATTGCTTAG
jb605c0380100.1
ATGCAGCAGCCAAGCACGATCGCCAGCACGCAGAGCGCTGCAAGCACAGCTTGGCTACGACCAGCGGCACGGCTCCCACGGCGCCGGCGGCA
CATGACAGCAGCCTCGGCAGCGGCGACCACCGAGCAGGCGCCTCGGCGTGTTGTGGTCACCGGCCAGGGCGTGGTCAGCAGCCTGGGGCACA
ACCCGGACGAATTCTACATGAACCTGCTAGCAGGGAAGAGCGGCATCAGCATGATTGACGGCTGGGACACAGAGGGTTACAGCACTCGTTTTGCG
GGGCAGATCAAGAGCCTGGACTGCGAGGGGTATGTGCCGCGGAAGTGGGAGAAGCGAATTGACGTGGTCATGAAGTACATGCAGGTGGCGGGG
AAGAAGGCCCTGGAGGATGCAGGCCTTCCCTGGGAAGGGCCAGAGCTGCAGGACTTGGACCGGCAGCGCTGCGGCATCTTGATAGGCACGGCC
ATGGGCGGCATGCAGACGTTTGCCAACGCCGTGGAGTCCCTGCAGACGAGCTTCAAGAAGATGAACCCCTTCTGCATTCCCTTTGCCATCCAGAA
CATGGGCGGTGCCATGCTGGCCATGGACCTTGGTTTCATGGGCCCCAACTACCCCATTGCAACCGCTTGCGCCACAGGCAACTACTGCATCCTCA
GTGCTGCCGACCACATTCGGCGAGGAGAGGCCGATCTGATGCTGGCCGGCGGTTCAGAGGCGGTCGTCATTCCCTCGGCCATTGCTGGCTTCAT
CGCCTGCAAAGCGCTGTCAAAGCGAAACGACGACCCTGCTGCTGCCAGCCGGCCTTGGGATCAGGGTCGTGACGGATTTGTGATGGGTGAAGG
CGCAGGTGTGTTGGTTCTGGAGGAGCTAGAGCATGCCAAGGCGCGGGGCGTGCCCATCCTGGCTGAGTTTGTGGGCGGCGACTTCACCTGCGA
CGCACACCACATGACTGAGCCCGAGCCAAACGGCCGAGGAGTCATCCTGTGCATTGAGCGAGCTCTGGCCAAGGCAGGGGTTGCTCCTGAAGAG
GTTGCATACGTAAATGCGCACGCCACCTCCACACCTGCTGGAGACATGGCTGAGTATCGAGCAATCACCACCGCACTGCCTCACAAGAGCCTGCG
AATCAACTCAACCAAGTCGATGATCGGCCACCTGCTGGGGGCGGCGGGAGCTGTGGAGGCGGTGGCCTCAATCCAGGCATTGCGCACAGGGTAC
CTGCACCCGAACCTCAACCTGGACAACCCTGAGGAGGCTGTGGATCTGAGTGTGGTGGTGGGGAGTGAGAAGCAGGAGTGGGACACCGAAGGA
GTTGTGCTGTCCAATTCCTTCGGGTTTGGCGGCCACAACAGCTGCATCATGTTTCGGAAGTTCAAGGGGTGA

Table S1. FA *de novo* synthase genes identified from JB6 .

**Gene name**	***Arabidopsis thaliana***	**JB6**
BCCP	AT5G15530.1	jb602c0162900.1
B-CT	ATCG00500.1	jb604c0327600.1
MCMT	AT2G30200.1	jb613c0887700.1
KAR	AT1G63380.1	jb601c0096200.1
KAS Ⅰ	AT5G46290.3	jb607c0563500.1
KAS Ⅱ	AT1G74960.2	jb605c0380100.1

Table S2. List of primers used for plasmid construction.

Primer Name	Primer Sequence (5'→3')
BCCP	F: CAACAAGCCCAGTTAACAGG ATGCAGGCAGCCATCACGGAGCGTG<br>R: AGGGCGGATGATCATCAGGGGCTGGTGCTCACCATGTACAGTAGG
B-CT	F: CAACAAGCCCAGTTAACAGG ATGAGCAGCAACGCAGGAGAGCTGG<br>R: CAGTGGAATATTGCCATGCTTGCGTTGCTCACCATGTACAGTAGG
MCMT	F: CAACAAGCCCAGTTAACAGG ATGCAGGCCGCCGCGATTCAGAGTG<br>R: CACAGTGATGTTCTCCAGGGGGTGGTGCTCACCATGTACAGTAGG
KAR	F: CAACAAGCCCAGTTAACAGG ATGTCGAAGCTTGTCGGGAAGGTCG<br>R: CATGGCTTCCTCCGGCACTTTGACGTGCTCACCATGTACAGTAGG
KAS Ⅰ	F: CAACAAGCCCAGTTAACAGG ATGTCACCAGCGCGTGTGCCGGAGG<br>R: AGCAATGTACTTTCCAAACGCGCACTGCTCACCATGTACAGTAGG
KASⅡ	F: CAACAAGCCCAGTTAACAGG ATGCAGCAGCCAAGCACGATCGCCA<br>R: CCCCTTGAACTTCCGAAACATGATGTGCTCACCATGTACAGTAGG

Table S3. List of primers used for qRT-PCR.

Primer Name	Primer Sequence (5'→3')
BCCP	F: TGCCCCAAGGTCGCTCGGTG<br>R: GAGTGCACCACTTAATCAGC
B-CT	F: AGGCAGCAATCCCAAGTGGG<br>R: GCTTGCCCTGCTTGTGCTGC
MCMT	F: GGCGGTCAAGGCCAGCTCTG<br>R: CCTTGGGGCCTTCACCGCAC
KAR	F: GGGCACGAGGCATTGGGCTG<br>R: CTTGCCCCGTCAAGAAGGTG
KAS Ⅰ	F: CGCGACAAGGGCTGCCCGCC<br>R: ATGCGAGGCCCATGCCAGTA
KASⅡ	F: GCAAGCACAGCTTGGCTACG<br>R: TGTAACCCTCTGTGTCCCAG
